# Supplementary figures and images for: Multi-scale immunoepidemiological modeling of within-host and between-host HIV dynamics: systematic review of mathematical models
Source: PeerJ. 2017 Sep 28;5:e3877. doi: 10.7717/peerj.3877 (PMC5623312; doi:10.7717/peerj.3877)

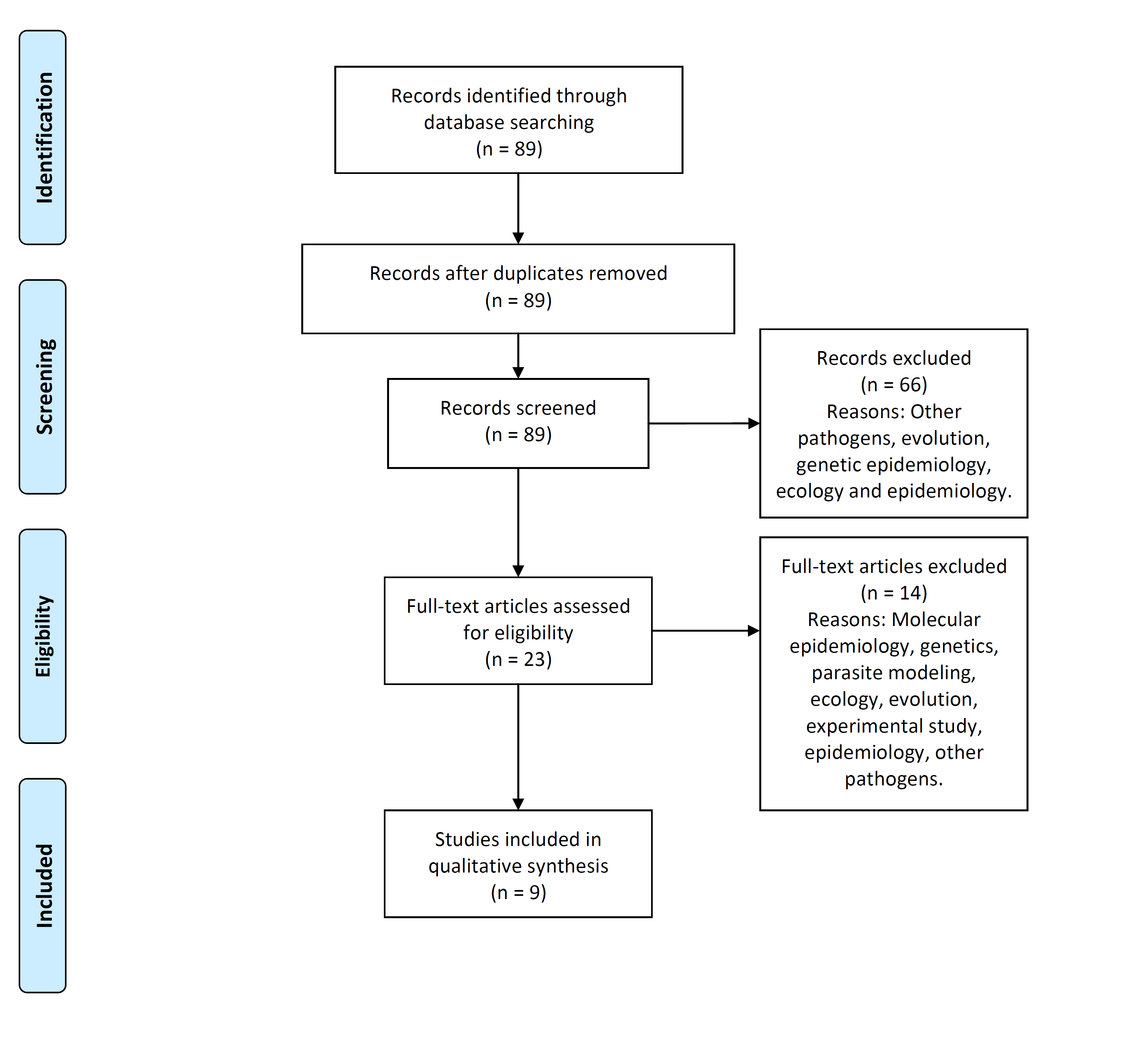

Supplement: Supplemental Information 1 [file peerj-05-3877-s001.png]
