# Supplementary material for: Multi-scale immunoepidemiological modeling of within-host and between-host HIV dynamics: systematic review of mathematical models
Source: PeerJ. 2017 Sep 28;5:e3877. doi: 10.7717/peerj.3877 (PMC5623312; doi:10.7717/peerj.3877)
Supplement: Supplemental Information 2 [file peerj-05-3877-s002.pdf]

PRISMA 2009 Checklist

Section/topic	#	Checklist item	Reported on page #
**TITLE**			
Title	1	Identify the report as a systematic review, meta-analysis, or both.<br>Excerpt: “Multi-scale Immunoepidemiological Modeling of Within-host and Between-host HIV Dynamics: Systematic Review of Mathematical Models”	1
**ABSTRACT**			
Structured summary	2	Provide a structured summary including, as applicable: background; objectives; data sources; study eligibility criteria, participants, and interventions; study appraisal and synthesis methods; results; limitations; conclusions and implications of key findings; systematic review registration number.<br>Excerpt: Background, objectives, methods, results, and conclusions in abstract	1-2
**INTRODUCTION**			
Rationale	3	Describe the rationale for the review in the context of what is already known.<br>Excerpt: “In this study, we review the multi-scale modeling methods that connect the within-host and between-host scales of HIV models. Understanding the relation between these two scales is key to understand HIV prognosis, transmission risk, and intervention effectiveness (Pepin et al. (2010)).”	4
Objectives	4	Provide an explicit statement of questions being addressed with reference to participants, interventions, comparisons, outcomes, and study design (PICOS).<br>Excerpt: “The objective of this study is to conduct a systematic review of multi-scale HIV immunoepidemiological models to infer the synergistic dynamics of HIV prognoses at the individual level and the transmission dynamics at the population level.”<br>Note: The PICOS framework is not applicable to this systematic review.	1
**METHODS**			
Protocol and registration	5	Indicate if a review protocol exists, if and where it can be accessed (e.g., Web address), and, if available, provide registration information including registration number.	NA
Eligibility criteria	6	Specify study characteristics (e.g., PICOS, length of follow-up) and report characteristics (e.g., years considered, language, publication status) used as criteria for eligibility, giving rationale.<br>Excerpt: “The inclusion criteria were articles focused on multi-scale immunoepidemiological modeling of HIV dynamics.”	5
Information sources	7	Describe all information sources (e.g., databases with dates of coverage, contact with study authors to identify additional studies) in the search and date last searched.<br>Excerpt: “We searched the PubMed database for articles published from December 1, 1985 to June 1, 2017…”	5

Search	8	Present full electronic search strategy for at least one database, including any limits used, such that it could be repeated.<br>Excerpt: "We searched the PubMed database for articles published from December 1, 1985 to June 1, 2017 with the terms: (HIV and ("multi-scale" or "immunoepidemiology" or "nested model" or ("within-host" and "between host") or ("within-host" and "among host") or ("within-host" and ("epidemiology" or "epidemiological")))).””	5
Study selection	9	State the process for selecting studies (i.e., screening, eligibility, included in systematic review, and, if applicable, included in the meta-analysis).<br>Excerpt: "During the identification step, articles were identified using the above search strategy. During the screening step, duplicate articles were removed, and titles and abstract of the remaining articles were screened to determine their relevance to our study. During the eligibility step, full texts of the articles were analyzed to determine their relevance to our study."	5
Data collection process	10	Describe method of data extraction from reports (e.g., piloted forms, independently, in duplicate) and any processes for obtaining and confirming data from investigators.	NA
Data items	11	List and define all variables for which data were sought (e.g., PICOS, funding sources) and any assumptions and simplifications made.	NA
Risk of bias in individual studies	12	Describe methods used for assessing risk of bias of individual studies (including specification of whether this was done at the study or outcome level), and how this information is to be used in any data synthesis.	NA
Summary measures	13	State the principal summary measures (e.g., risk ratio, difference in means).	NA
Synthesis of results	14	Describe the methods of handling data and combining results of studies, if done, including measures of consistency (e.g., I^2) for each meta-analysis.	NA
Risk of bias across studies	15	Specify any assessment of risk of bias that may affect the cumulative evidence (e.g., publication bias, selective reporting within studies).	NA
Additional analyses	16	Describe methods of additional analyses (e.g., sensitivity or subgroup analyses, meta-regression), if done, indicating which were pre-specified.	NA
**RESULTS**			
Study selection	17	Give numbers of studies screened, assessed for eligibility, and included in the review, with reasons for exclusions at each stage, ideally with a flow diagram.<br>Excerpt: Figure 2	26
Study characteristics	18	For each study, present characteristics for which data were extracted (e.g., study size, PICOS, follow-up period) and provide the citations.<br>Excerpt: Table 1	28-29
Risk of bias within studies	19	Present data on risk of bias of each study and, if available, any outcome level assessment (see item 12).	NA
Results of individual studies	20	For all outcomes considered (benefits or harms), present, for each study: (a) simple summary data for each intervention group (b) effect estimates and confidence intervals, ideally with a forest plot.	NA

PRISMA 2009 Checklist

Synthesis of results	21	Present results of each meta-analysis done, including confidence intervals and measures of consistency.	NA
Risk of bias across studies	22	Present results of any assessment of risk of bias across studies (see Item 15).	NA
Additional analysis	23	Give results of additional analyses, if done (e.g., sensitivity or subgroup analyses, meta-regression [see Item 16]).	NA
**DISCUSSION**			
Summary of evidence	24	Summarize the main findings including the strength of evidence for each main outcome; consider their relevance to key groups (e.g., healthcare providers, users, and policy makers).<br>Excerpt: "Table 9 illustrates the clinical and public health relevant problems of HIV virulence, co-infection, super-infection, drug resistance and treatment dynamics that can be potentially addressed using multi-scale models…"<br>Table 1	16, 28-29
Limitations	25	Discuss limitations at study and outcome level (e.g., risk of bias), and at review-level (e.g., incomplete retrieval of identified research, reporting bias).<br>Excerpt: "We reviewed English language articles on HIV immunoepidemiological models that were referenced in the PubMed database. The dynamics of the HIV immunoepidemiological models are dependent on the selection of parameters, and the coupling mechanisms of within-host immune-viral dynamics and between-host transmission dynamics. Verification and validation of HIV immunoepidemiological models (and multi-scale models in general) with empirical data is a challenge to be addressed in future studies. Also, the selection of optimal layers from the genomic, molecular, cellular, and organ levels at the micro-biological scale to the individual, family, community, national, and global levels at the macro-social scale is a challenge that need be addressed well in future studies."	17
Conclusions	26	Provide a general interpretation of the results in the context of other evidence, and implications for future research.<br>Excerpt: "HIV immunoepidemiological models combine the immune-viral dynamics at the within-host immunological scale with the transmission dynamics at the between-host epidemiological scale to analyze HIV dynamics of a single strain infection, co-infection, super-infection, evolution, drug resistance, and treatment protocols in heterogeneous populations. Based on our understanding of synergistic dynamics of HIV at the individual and population scales, we should select the optimal layers of analysis from micro-biological to macro-social levels for multi-scale models to identify and improve solutions to clinical and public health relevant problems of HIV dynamics."	17
**FUNDING**			
Funding	27	Describe sources of funding for the systematic review and other support (e.g., supply of data); role of funders for the systematic review.	NA
