# Supplementary material for: Multi-scale immunoepidemiological modeling of within-host and between-host HIV dynamics: systematic review of mathematical models
Source: PeerJ. 2017 Sep 28;5:e3877. doi: 10.7717/peerj.3877 (PMC5623312; doi:10.7717/peerj.3877)
Supplement: Supplemental Information 3 [file peerj-05-3877-s003.pdf]

1. **The rationale for conducting the systematic review.**

   The rationale for conducting the systematic review of multi-scale HIV immunoepidemiological models is to infer the synergistic dynamics of HIV prognoses at the individual level and the transmission dynamics at the population level.

2. **The contribution that the systematic review makes to knowledge in light of previously published related reports, including other meta-analyses and systematic reviews.**

   HIV immunoepidemiological models connect the within-host immune dynamics at the individual level and the epidemiological transmission dynamics at the population level. While multi-scale models add complexity over a single-scale model, they account for the time varying immune viral response of HIV+ individuals, and the corresponding impact on the time-varying risk of transmission of HIV+ individuals to other susceptibles in the population. They are useful to analyze the dynamics of HIV super-infection, co-infection, drug resistance, evolution, and treatment in HIV+ individuals, and their impact on the epidemic pathways in the population. We illustrate the coupling mechanisms of the within-host and between-host scales, their mathematical implementation, and the clinical and public health problems that are appropriate for analysis using HIV immunoepidemiological models.
